# A case report of long-delayed diagnosis of pseudorabies virus encephalitis with endophthalmitis: lessons from metagenomic next generation sequencing

**DOI:** 10.1186/s12883-023-03227-1

**Published:** 2023-05-16

**Authors:** Yi Zhang, Lei Fang, Yi Zhou, Yongqing Zhang, Bing Liang, Chuanzhu Yan, Ling Li

**Affiliations:** 1 Department of Neurology, Qilu Hospital(Qingdao), Cheeloo College of Medicine, Shandong University, 758 Hefei Road, Qingdao, 266035 Shandong China; 2 Department of Neurology, Rizhao Central Hospital, 66 Wanghai Road, Rizhao, 276800 Shandong China; 3 Department of Neurology, Qingdao Central Hospital, No.127, Siliu South Road, Qingdao, Shandong China; 4 Department of Ultrasonic, Rizhao Hospital of Traditional Chinese Medicine, 35 Wanghai Road, Rizhao, 276800 Shandong China

**Keywords:** Pseudorabies virus, Viral encephalitis, Endophthalmitis, Next generation sequencing, Antiviral therapy

## Abstract

**Background:**

Pseudorabies virus (PRV) was thought to only infect animals. Recent studies have shown that it can also infect human.

**Case presentation:**

We report a case of pseudorabies virus encephalitis and endophthalmitis, diagnosed 89 days after onset, confirmed with intraocular fluid metagenomic next generation sequencing (mNGS) after the result of two cerebrospinal fluid (CSF) mNGS tests were negative. Although treatment with intravenous acyclovir, foscarnet sodium, and methylprednisolone improved the symptoms of encephalitis, significant diagnostic delay resulted in permanent visual loss.

**Conclusions:**

This case suggests that pseudorabies virus (PRV) DNA in the intraocular fluid may have a higher positivity than that in the CSF. PRV may persist in the intraocular fluid for an extended period and may thus require extended antiviral therapy. Patients with severe encephalitis and PRV should be examined with the focus on pupil reactivity and light reflex. A fundus examination should be performed in patients with a central nervous system infection, specifically, those in a comatose state, to help reduce eye disability.

**Supplementary Information:**

The online version contains supplementary material available at 10.1186/s12883-023-03227-1.

## Introduction

Pseudorabies virus (PRV) is a member of the Herpesviridae family, Alphaherpesvirinae subfamily, and Varicellovirus genus. PRV is also known as Suid herpes virus type 1(SHV1) and is the causative agent of Aujeszky’s disease.PRV is a neurotrophic alpha herpes virus with a double-stranded DNA genome. Swine are the main natural hosts and sources of PRV infection. PRV spreads via direct contact although transmission may occur via water, air, and contaminated fomites. PRV dose have the ability to cross the placenta and infect animals in utero, leading to reproductive failure in infected animals, such as sows [1]. Although maternal IgG provides protection, high doses of PRV virus can overcome low maternal IgG [2]. Moreover, PRV can cause fatal encephalitis associated with a mortality rate of 100%; it may also cause respiratory disorders in growing-fattening pigs, and reproductive failure in sows [3]. In addition, PRV infects a wide range of other hosts, including cattle, sheep, dogs, cats, chickens, rodents, rabbits, foxes, and some non-human primates, suggesting PRV can cause cross species transmission [4]. The virus was first identified in humans in 1914; however, these cases lacked evidence such as antibodies or PRV sequences. In 1987, Mravak et al. reported on three patients with suspected human SHV1 infection, confirmed by test results positive for the SHV1 antibody [5]. In 2018, Ai et al. reported a case of human endophthalmitis caused by PRV infection, which was confirmed by PRV-specific sequences in the patients’ vitreous humor by metagenomic next-generation sequencing(mNGS) [6]. In 2019,the first case of PRV resulting in acute, fulminating, and fatal central nervous system infection, was reported [7]. Herein, we report a human case of PRV encephalitis and endophthalmitis diagnosed with mNGS of the aqueous humor approximately 89 days after onset. Diagnosis was confirmed with intraocular fluid mNGS after results of two cerebrospinal fluid (CSF) mNGS tests were negative.

## Case report

Patient has provided informed consent for publication of the case. The study was performed in accordance with the code of ethics of the world medical association (declaration of Helsinki) for experiments involving humans and the protocol was approved by the research ethics committee of Qilu Hospital of Shandong University.

**Fig. 1:**
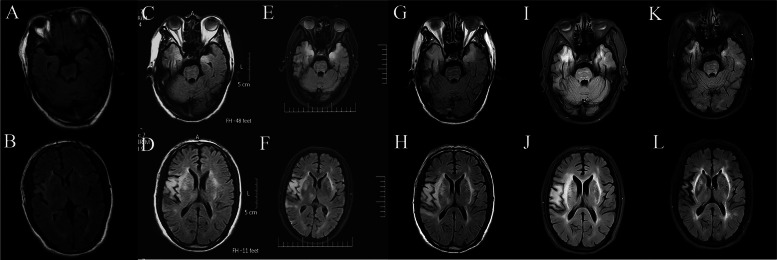
The plain brain MRI at different time after onset [(A.B) 9th day, (C.D) 48th day, (E.F) 54th day; (G.H) 69th day;(I.J)94 day; (K.L)154 day;] after initial symptom. (A.B) Brain MRI -FLAIR scans revealed hyperintense signal changes in the right frontal lobe, insula cortex. (C.D) New lesions appeared in the right temporal lobes, left hippocampus, bilateral insular lobe, brain stem and right basal ganglia at the 48th day after initial symptoms. (E.F) FLAIR analysis show compared with Figure C.D, there was also a new lesion in the right thalamus at the 54th day. (G.H) In contrast to Figure E.F, the lesions in the bilateral temporal and insula are enlarged at the 69th day. (I.J) There was no further enlargement of the lesion at the 94^th^ day.(K.L) On day 154 after the onset, the patient's symptoms improved, and follow-up MRI showed that all lesions were significantly reduced

A 56-year-old man was injured when his hands were cut by a knife used to carve dead swine. He had no pre‐existing medical conditions and was employed as a butcher in Shandong Province, China. Four days after the injury, he lost his appetite, developed a fever of 37.7 °C, and reported fatigue. Nine days after the injury, he was admitted to a local emergency center, following a tonic–clonic seizure. A routine blood test revealed increased neutrophil counts, and mildly elevated liver enzyme levels. (supplementary table 1). Computed tomography (CT) scan findings were normal; however, the patient continued to have seizures and eventually became comatose. The Glasgow Coma Scale (GCS) score was E1V1M2. The patient was transferred to the intensive care unit (ICU)after an endotracheal intubation and treated with intravenous midazolam and levetiracetam to control the seizures. A CSF sample was obtained, presenting as colorless and clear with a pressure of 180 mmH2O (80–180 mmH2O). The CSF analysis revealed 0 cells /mm3(normal range 0–5 cells/mm3). Bacterial, fungal, and tuberculosis CSF cultures were negative along with mNGS. Blood glucose was 4.34 mmol/L (normal range 2.8–4.5 mmol/L), chloride 126.0 mmol/L (normal range 120–130 mmol/L), and protein 0.595 g/L (normal range 0.15–0.45 g/L). Brain magnetic resonance imaging (MRI) and fluid‐attenuated inversion recovery (FLAIR) scans revealed hyperintense signal changes in the right frontal lobe, insula cortex (Fig. 1A, B). The local hospital suspected viral encephalitis and administered antiviral therapy (acyclovir 0.5 g, q8h) with continued intravenous administration of midazolam and sodium valproate (VPA) to relieve seizures.

On day 11, the patients’pupil was dilated to 5 mm and non‐reactive. Lumbar puncture revealed normal CSF pressure. The CSF analysis revealed 25 leukocytes/μL (including 96% mononuclear cells), and 725.3 mg/L of protein. Glucose and chloride levels were normal. Both the CSF smear and culture were negative (supplementary table 1). Further CSF analysis was negative for autoimmune encephalitis-related antibodies; moreover, CSF mNGS findings were negative. The patient remained in the ICU and received treatment with methylprednisolone (500 mg/day) for 3 days, intravenous immunoglobulin (IVIg) (0.4 g/kg/day) for 5 days, and anticonvulsive medication, including midazolam (0.05 mg/kg/h), sodium valproate (800 mg, q12h), and phenobarbital sodium (100 mg, q12h). Repeat brain MRI revealed that new lesions in the right temporal lobes, left hippocampus, bilateral insular lobe, brain stem and right basal ganglia (Fig. 1C, D).

On day 51, the patient regained consciousness and was responsive, although. He reported being blind. An ophthalmic consultation revealed extensive pupil adhesions on both sides, iris surface neovascularization, and lens opacity. The fundus could not be observed and binocular uveitis was diagnosed.

The seizures ceased after VPA treatment. FLAIR analysis revealed hyper-intensity in the right temporal lobe, bilateral basal ganglia, bilateral hippocampus, brain stem and bilateral lateral paraventricular region (Fig. 1E, F). The patient was discharged to a rehabilitation facility, progressively recovering the capacity to walk.

**Fig. 2:**
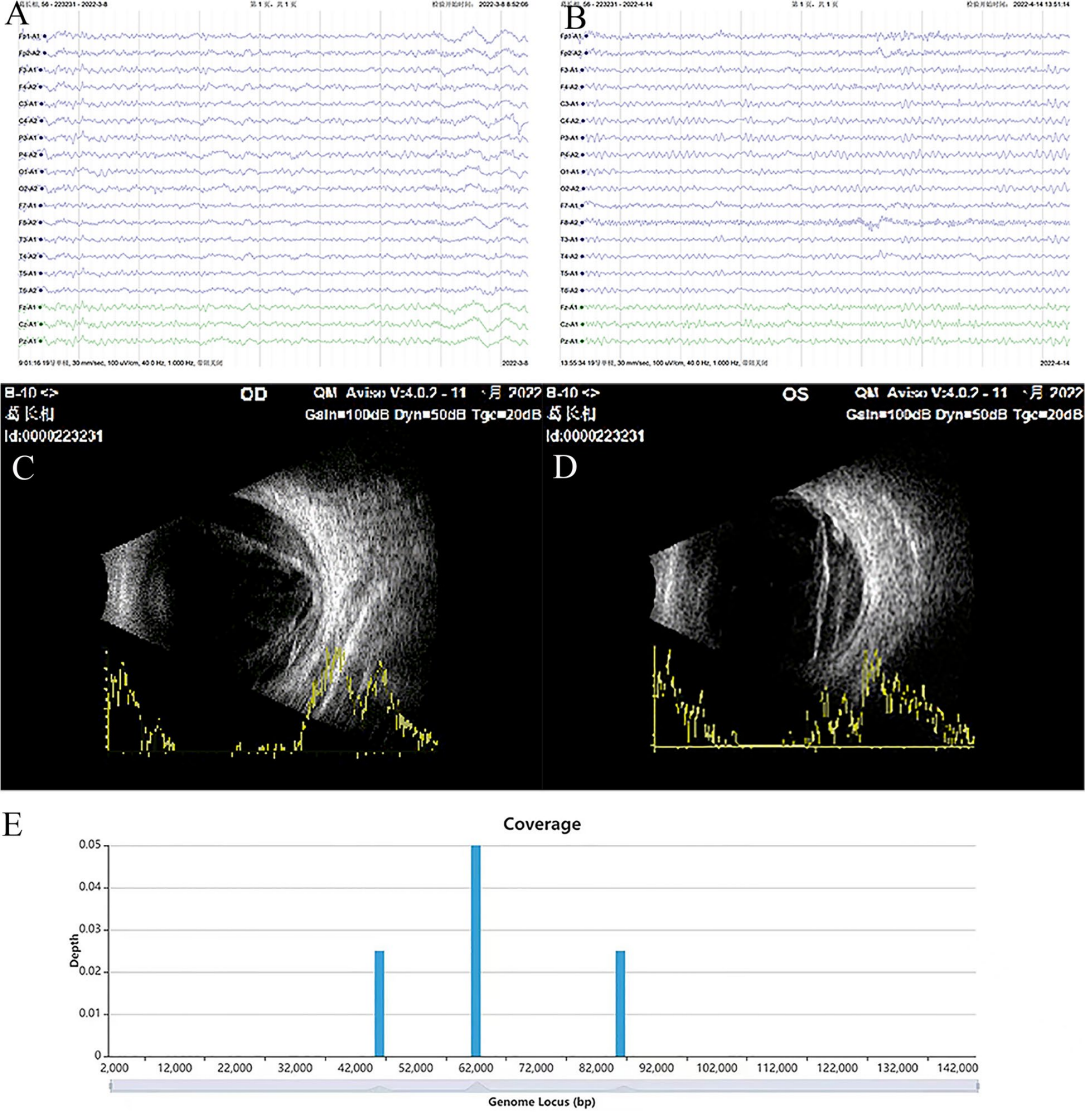
**A** Electroencephalogram (EEG) showed diffuse slow waves. **B **Repeat electroencephalogram(EEG) findings were generally normal. **C**.**D **Binocular ultrasound findings suggested retinal detachment in both eyes. **E** the mNGS results showed a total of four PRV reads in the aqueous humor, accounting for 0.02% of the whole genome

On day 63, the patient’s body temperature reached 38.1 ℃. The seizures recurred at a frequency of 3 per day, lasting 3–5 min per episode. The patient also presented with hyperkinetic movement whereby the limbs wriggled uncontrollably. The patient was transferred to our hospital. Physical examination revealed left and right pupil dilation of 3 and 4 mm, respectively, without light reflex or light perception. Meanwhile, bilateral upper limb muscle tone was high, with left and right-side muscle strength levels of 3 and 4 points, respectively. Involuntary twisting and negative Babinski signs were observed bilaterally. Repeat CSF examination revealed 9 cells /mm3 with lymphocytic predominance (90%), and 0.79 g/L of protein (normal range 0.15–0.45 g/L), 155.74 mg/l of IgG (normal range 0–34 mg/l), and 25.49 mg/l of IgA (normal range 0–5 mg/l). However, both CSF and antibody tests remained negative for autoimmune encephalitis. The abnormal FLAIR signal increased symmetrically in the brain stem, bilateral basal ganglia, and external capsule (Fig. 1G, H). Binocular ultrasound findings suggested retinal detachment in both eyes (Fig. 2C, D). Electroencephalogram (EEG) showed diffuse slow waves (Fig. 2A). Given the clinical presentation and test results, the differential diagnosis was viral encephalitis, which was treated with methylprednisolone (0.5 g/d for 3 days then decreading to 0.25 g/d for 3 days) and antiviral acyclovir (10 mg/kg, every 8 h for 21 days). Treatment included antiepileptic drugs such as levetiracetam, valproic acid, and clonazepam for movement disorder. To identify the causative virus, on day 85, the ophthalmologist used a 1 mL syringe needle to puncture the anterior chamber from the limbus of the cornea and extracted 0.3 mL aqueous humor, after surface anesthesia and local disinfection. We analyzed 0.3 ml of aqueous humor and 2 ml of the CSF with metagenomic next-generation sequencing.

**Fig. 3:**
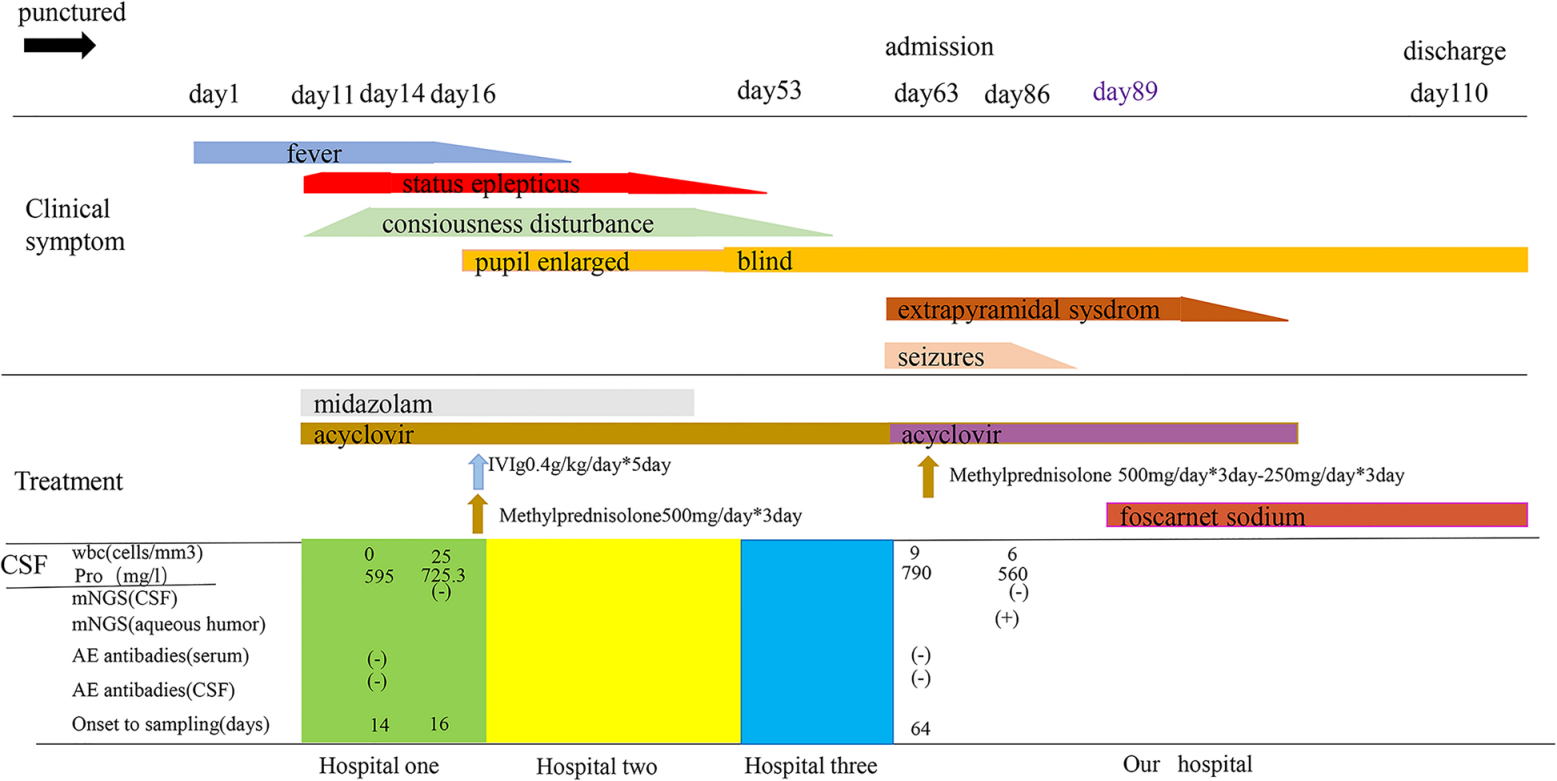
Schematic representation of clinical course. On day 89, the mNGS of the aqueous humor discovered PRV nucleic acid. We started treatment with foscarnet sodium and the patient’s clinical symptoms improved gradually. Different colors represent treatment at different hospitals in last column

On day 89, the mNGS results revealed a total of four PRV reads in the aqueous humor, accounting for 0.02% of the whole genome (Fig. 2E). However, the CSF mNGS test results were negative. PRV encephalitis with endophthalmitis was diagnosed. Subsequently treatment with foscarnet sodium (6 g/day, every 8 h, for 28 days) was initiated, an antiviral specific for herpes virus infection. After the administration of foscarnet sodium, clinical symptoms gradually improved (Fig. 3). Repeat MRI revealed abnormal signals in the brain stem, bilateral basal ganglia, and external capsule (Fig. 1I, J). Repeat EEG findings were generally normal (Fig. 2B).

On day 110, the patient was discharged. On day 154, a follow-up brain MRI revealed reduced lesions (Fig. 1K, L). Despite bilateral blindness and hand tremors, epileptic seizures did not recur, and the patient could walk independently.

**Fig. 4:**
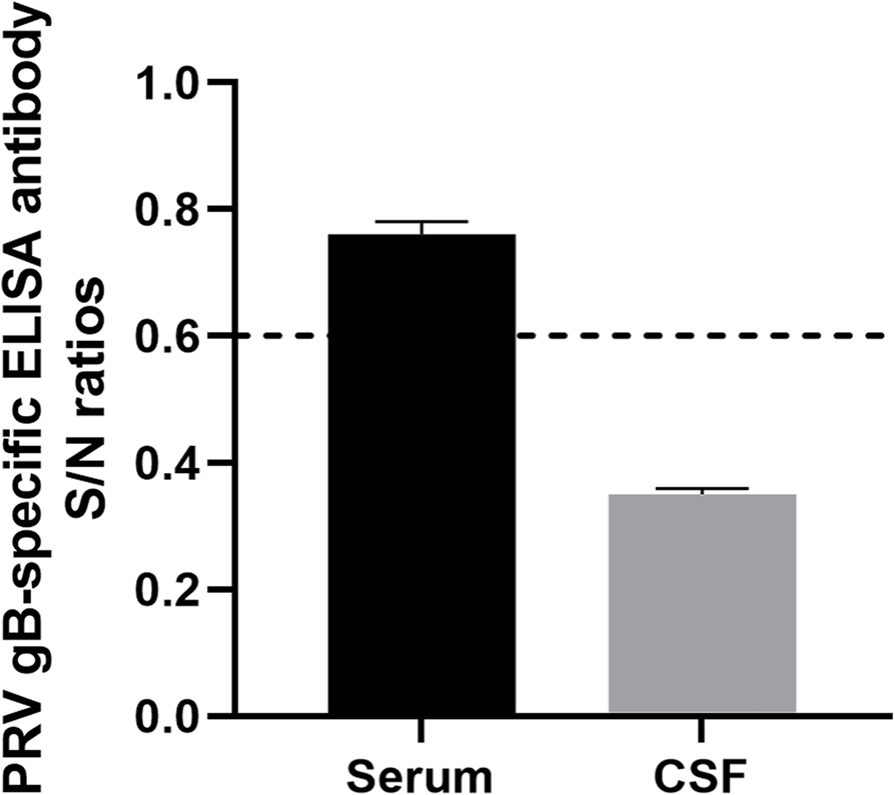
The PRV gB-specific antibody of the encephalitis patient. The dashed line serves as the critical value. S/N value greater than 0.6 indicates a negative result, while S/N value less than 0.6 indicates a positive result

For the reads of PRV in mNGS were only 4, we defrosted the patient’s serum and CSF frozen(-80℃) on day 85, and performed the PRV antibody testing in Sichuan Agricultural University later. PRV antibody was detected in serum (Fig. 4 and Table 1).

**Table 1:** The result of PRV gB-specific antibody in the serum and CSF samples

Samples	OD450	Positive	Negative	S/N ratio
CSF	0.869	0.086	1.179	0.74
	0.917			0.78
	0.893			0.76
Serum	0.398			0.34
	0.411			0.35
	0.429			0.36

### mNGS

The collected atrial fluid samples were detected by Dian Diagnostics Group Co., Ltd. (Hangzhou, China) for pathogenic next-generation metagenomic sequencing (mNGS). The samples were added to an NGS master automated workstation for automated nucleic acid extraction, reverse transcription (RNA-only), nucleic acid fragmentation, end-complementation, terminal adenylation (single base A at the 3'-end), sequencing adapter ligation, and purification to form a sequencing library. Libraries were quantified by fluorescent quantitative PCR and then sequenced by shotgun sequencing using the Illumina Nextseq platform (California, USA). Each library yielded an average of 20 million single-end 50 bp sequence data per read. The raw sequencing data were filtered and the remaining microbial sequence data were compared to reference databases (NCBI GenBank and local microbial genome data of Dian Diagnostics Group Co., Ltd.) to determine microbial species and relative abundance. A negative control (a mixture of plasma-free nucleic acids and fragmented human genomic DNA) and a positive control (a mixture containing inactivated bacterial, fungal and pseudoviral particles) were added to each round of mNGS testing. The mNGS reporting rules: firstly, the sequence data meet QC requirements (library concentration > 50 pM, Q20 > 85%, Q30 > 80%); secondly, the negative control (NC) has no detection of the species in the same microarray or an RPM (sample)/ RPM (NC) ≥ 5, as a threshold for distinguishing true positives from background contamination threshold.

Wet lab procedures to prevent contamination: For each sequencing run, a negative control (culture medium containing 104 Jurkat cells/mL) was included. Dry lab procedures to prevent or identify contamination: Microbial reads identified from a library were reported if: 1) the sequencing data passed quality control filters (library concentration > 50 pM, Q20 > 85%, Q30 > 80%); 2) negative control (NC) in the same se-quencing run does not contain the species or the RPM (sample) / RPM (NC) ≥ 5, which was determined according to previous studies as a cutoff for discriminating true-positives from background contaminations.

## Discussion

In 2017, Li et al. suggested that PRV may interact with human and swine cellular receptor nectin-1 via envelope glycoprotein D on the viral surface, indicating a PRV variant may infect humans [8]. To our knowledge, there have been 25 cases of PRV encephalitis or endophthalmitis (Table 2); among these,14 (case3,4,6,7,10,12,17,19–25) of the 25 cases involved the eye [6, 9–16]. Among the cases with ocular involvement, only six cases (case17,20–24) underwent second-generation sequencing of the intraocular fluid [6, 11, 13–15], in which DNA fragments of PRV were detected, confirming human pseudorabies virus-related endophthalmitis at the DNA level. PRV DNA was detected in the intraocular fluid of patients at 11, 16, 23, and 25 days, and at 3 and 4 months after symptom onset.

**Table 2:** Current status of pseudorabies virus infection in human

Case	Reference	Encephalitis		Endophthalmitis					Cerebrospinal fluid (mNGS)		Intraocular fluid(mNGS)		Treatment	Other organs
		Y/N	Outcome	Y/N	Optic examination	Pupil dilation	Light reflex	Outcome	Neg/Pos	Onset to sampling (D)	Neg/Pos	Onset to sampling(D)		
1	Zhou etal [17]	Y	Unconsciousness	N	ND	ND	ND	ND	Pos	14	ND	ND	ACV	1
2	Zhou etal. [17]	Y	Died	N	ND	ND	ND	NA	Pos	11	ND	ND	ACV + IVIG	1
3	Fan etal [9]	Y	Died	Y	Funduscopy	ND	ND	NA	Pos	7	ND	ND	ACV + IVIG + MPS	1 + 2
4	Fan etal [9]	Y	m RS 3	Y	Funduscopy OCT	ND	ND	Improved	Pos	4	ND	ND	ACV + PFAt + MPS	1 + 2
5	Fan etal [9]	Y	m RS 3	N	ND	Dilation	Disappear	ND	Pos	2	ND	ND	ACV + IVIG	1 + 2
6	Fan etal [9]	Y	Died	Y	Funduscopy	ND	ND	NA	Pos	2190	ND	ND	ACV + Foscarnet	1 + 2
7	Fan etal [9]	Y	Died	Y	Funduscopy	Irregular	Slow	ND	Pos	2	ND	ND	ACV + IVIG + GC	1 + 2
8	Fan etal [9]	Y	died	N	N	Equal	Sensitive	ND	Pos	4	ND	ND	ACV + MPS	1 + 2
9	Fan etal [9]	Y	mRS 5	N	N	Equal	Sensitive	ND	Pos	4	ND	ND	ACV + IVIG + MPS	1 + 2
10	Liu etal [10]	Y	Improved	Y	Fundus images OCT	ND	ND	Blindness	Pos	6	ND	ND	PFA	ND
11	Liu etal [10]	Y	Improved	N	ND	ND	ND	ND	Pos	7	ND	ND	PFA	1
12	Liu etal [10]	Y	Unconsciousness	Y	ND	ND	ND	ND	Pos	9	ND	ND	PFA	1
13	Yang etal [7]	Y	Unconsciousness	Y	ND	ND	ND	ND	Pos	8	ND	ND	RBV + MPS + IVIG	1 + 3 3
14	Wang etal [18]	Y	Improved	N	ND	ND	ND	ND	Pos	5	ND	ND	ACV + GC	1
15	Yang etal [11]	Y	mRS 4	N	ND	ND	ND	ND	Pos	5	ND	ND	IVIG + GC + Antiviral	2
16	Yang etal [11]	Y	Unconsciousness	N	ND	ND	ND	ND	Pos	9	ND	ND	IVIG + GC + Antiviral	2
17	Yang etal [11]	Y	mRS 3	Y	Funduscopy	ND	Disappear	Blurry vision/Recurrence	Pos	10	Pos	120	IVIG + GC + ACV + Eye surgery	2
18	Yang etal [11]	Y	Unconsciousness	N	ND	ND	ND	ND	Pos	5	ND	ND	IVIG + GC + antiviral	2
19	Yang etal [11]	Y	mRS 3	Y	Funduscopy Ultrasound	ND	ND	Blindness/Recurrence	Pos	12	ND	ND	IVIG + GC + Antiviral	2 + 4
20	Hu etal [12]	Y	Improved	Y	Fundus images Ultrasound	ND	ND	Blindness/Recurrence	Pos	90	Pos	ND	ACV + Eye surgery	ND
21	Yan etal [13]	Y	Unconsciousness	Y	ND	3 mm	L-sluggishly R-disappear	ND	Pos	11	Pos	11	PFA + ACV + MPS	2
22	Ying etal [14]	Y	Improved	Y	OCT FFA	ND	Sluggishly	Impaired vision	Pos	8	Pos	23	GCV + PFA	ND
23	Ai etal [6]	N	Improved	Y	Fundus images OCT	ND	Sluggishly	Impaired vision	Neg	NA	Pos	16	Vitrectomy + VCV	ND
24	Liu etal [15]	Y	mRS 3	Y	Fundus images Ultrasound	Dilation	Disappear	Blindness	Pos	18	Pos	2	IVIG + GCV + MPS	ND
25	Liu etal [16]	Y	Improved	Y	Fundus images Ultrasound	ND	Sluggishly	Impaired vision	Pos	2/41/54	ND	ND	GCV + ACV + PFA + GC	ND

*L* Left, *R* Right, *mRS* Modified Rankin Scale, *ND* No description, *Neg* Negative, *Pos* Positive

Optic examination: *FFA* Fundus fluorescein angiography, *OCT* Optical coherence tomography

Treatment: *ACV* Acyclovir, *VCV* valacyclovir, *GCV* ganciclovir, *PFA* Phosphine formic acid/Foscarnet; *RBV* Ribavirin, *MPS* Methylprednisolone, *GC* glucocorticoids, *IVIG* intravenous immunoglobulin

Other organs: 1 = respiratory failure; 2 = Pneumonia;3 = gastrostomy tubes; 4 = peptic ulcer

Herein, we have presented a case of PRV encephalitis complicated by endophthalmitis. The diagnosis was confirmed only on day 89 after symptom onset using mNGS of the intraocular fluid. Previous mNGS results of CSF had been negative. These findings suggest clinicians should accurately test all the affected body fluids, not just CSF, to increase the probability of having a positive result and identifying PRV infection. In cases with ocular involvement, the aqueous humor and vitreous fluid may be used in mNGS, which could help achieve early diagnosis. We speculate that PRV may have greater survival in the intraocular fluid than in the CSF, as previously observed [14]. The prolonged presence of the virus in the eye suggests that clinicians should routinely monitor both the CSF and intraocular fluid with mNGS during the management of patients with PRV encephalitis or endophthalmitis. If PRV nucleic acid test results remain positive, the duration of antiviral treatment should obviously be extended, helping reduce the risk of viral reactivation, re-replication, or exacerbated inflammation caused by inadequate antiviral treatment. Improvement of clinical symptoms may also be a treatment endpoint.

In previous case reports, two(case5,14) of 11(case1,2,5,8,9,11,13–16,18) PRV encephalitis patients with unreported endophthalmitis had pupil changes and light reflex loss 7,9,10,11,12 [7, 9–11, 18, 19]. We suspect these patients experienced intraocular infections, which were not confirmed due to patient comatose status. Only six (case17,case21-25) of 14(case3,4,6,7,10,12,17,19,20–25) endophthalmitis cases reported pupil or light reflex abnormalities. One patient with endophthalmitis had symptoms comparable to those observed in the present patient, including dilated pupils in a coma state, followed by awakening with reduced vision. During treatment at a local hospital, the present patient was observed to have dilated pupils and light reflex loss, which were misattributed to aggravated encephalitis. However, the patient’s vision loss upon waking from the coma might have been attributable to ocular inflammation; eye examination revealed retinal detachment. These findings suggest that clinicians should evaluate eye lesions in patients suspected of having PRV infection, regardless of their coma status. Cases involving enlarged pupils and reduced light reflexes should undergo fundus examination, which may reveal abnormalities that should be treated locally without delay. In patients with a central nervous system infection, especially when comatose, the fundus examination should be performed routinely, and supplemented with ocular ultrasound as needed. This is to detect and treat possible ocular lesions promptly, even with no change in the response to light. Local injection of penciclovir sodium, phosphopotassium, and dexamethasone may help treat endophthalmitis caused by PRV [14], helping reduce morbidity and mortality rates.

Endophthalmitis is the characteristic clinical manifestation of pseudorabies virus encephalitis. However, the exact mechanism by which PRV causes human eye infection remains unclear. In the mammalian model of PRV entry, gD epitope on the surface of PRV can bind to specific cellular receptors such as herpesvirus entry mediator (HVEM) and nectin-1 to stabilize the virion-cell interaction [17]. Nectin-1 is predominantly expressed in the nervous system [20, 21]. HVEM is widely expressed in retinal pigment epithelial cells, conjunctival and corneal epithelial cells, corneal fibroblasts and trabecular meshwork cells [22]. This may be the key for PRV to enter the eye tissue, promoting ocular inflammation and causing neovascularization, corneal scarring and thinning, retinitis and retinal detachment [23].

In the present case, viral encephalitis and autoimmune encephalitis were considered at the local hospital. Antiviral therapy was initiated early, with methylprednisolone and gamma globulin administered. The patient's clinical symptoms gradually improved, and he regained the capacity to walk with assistance. However, 63 days thereafter, the patient developed recurrent seizures with extrapyramidal movements and fever. Repeat brain MRI scans revealed enlarged lesions (Fig. 1G.H), while mNGS of an aqueous humor sample detected PRV DNA. The recurring seizures were likely the result of virus reactivation and replication, triggered by fever, after the incomplete antiviral treatment. A previous study reported the risk of an immune inflammatory response during HSV primary infection and upon reactivation. An uncontrolled immune inflammatory response may promote disease progression [24]. Symptoms may improve or stabilize after treatment with steroids [25, 26]. PRV and herpes simplex virus are both alpha herpes viruses with similar biological characteristics; in both cases, the treatment may involve antiviral and hormonal therapies.

In contrast to the present case, three (case17,19,20)of 14(case3,4,6,7,10,12,17,19–25) patients with PRV endophthalmitis had recurrent vision loss after PRV encephalitis improvement (Table 2), including two cases in whom PRV DNA was detected. The induction of endophthalmitis in these patients may have been due to the reactivation of latent PRV within the brain, which progressed to the eye. In a mammalian model, PRV establishes lifelong latency in the trigeminal ganglia (TG) [27]. The same as HSV, PRV particles may be reactivated by fever or other stress, and invade the central nervous system, the eyes, skin and mucosa via anterograde or retrograde transsynaptic pathways, [28]. Moreover, patients with herpes simplex encephalitis may induce autoimmune encephalitis [24, 29]. PRV encephalitis may involve similar mechanisms. While, antibody tests were negative for autoimmune encephalitis in our case. Whether PRV encephalitis can induce autoimmune encephalitis remains unclear.

In a word, based on the present findings, we recommend timely fundus examination for all patients suspected of having PRV or other severe encephalitis. PRV eye infection should be found as early as possible, so that local eye treatment can be promptly started. Specifically, in cases that involve pupil and light reflex changes. Even if the CSF mNGS findings are negative, mNGS of the intraocular fluid or other tissues should be performed repeatedly. A antiviral therapy should be continued if the PRV nucleic acid test results are positive. Finally, although an improvement of clinical symptoms may indicate virus elimination, the virus may remain latent for prolonged periods. Consequently, lifelong follow-up evaluations are required for affected patients, including eye infection assessments, to help reduce recurrence and mortality.

This case study had some limitations. First, as follow-up clinical and MRI findings suggested symptom improvement, mNGS of pre-aqueous humor was not reviewed. Second, our understanding of the mechanisms involved in human PRV infections remain incomplete and thus require further research. Third, although cell based array (CBA) of any known autoimmune antibody is negative, we did not complete tissue based assay (TBA) of the CSF was not evaluated to determine whether the patient had autoimmune encephalitis. Then, because of sample unitability, we cannot perform other tests such as antigen testing or PCR, or proof of infection by antibody testing.

## Supplementary Information

**Additional file 1.**

## Data Availability

The raw data of microbial macrogenome sequencing has been uploaded to the Genome Sequence Archive (GSA), which strictly follows the data standards of the INSDC consortium. The names of the repository/repositories and accession number(s) can be found at: https://bigd.big.ac.cn/gsa/browse/CRA009108, CRA009108.

## Abbreviations

PRV: Pseudorabies virus
mNGS: Next generation sequencing
CSF: Cerebrospinal fluid
SHV1: Suid herpes virus type 1
CT: Computed tomography
ICU: Intensive care unit
MRI: Brain magnetic resonance imaging
FLAIR: Fluid‐attenuated inversion recovery
VPA: Sodium valproate
IVIg: Intravenous immunoglobulin
EEG: Electroencephalogram
HSV: Herpesvirus hominis
HVEM: Herpesvirus entry mediator
TG: The trigeminal ganglia
CBA: Cell based array
TBA: Tissue based assay

## References

[1] Iglesias JG, Harkness JW (1988). Studies of transplacental and perinatal infection with two clones of a single Aujeszky's disease (pseudorabies) virus isolate. Vet Microbiol.

[2] Paul PS, Halbur P, Janke B, Joo H, Nawagitgul P, Singh J (2003). Exogenous porcine viruses. Curr Top Microbiol Immunol.

[3] Pomeranz LE, Reynolds AE, Hengartner CJ (2005). Molecular biology of pseudorabies virus: impact on neurovirology and veterinary medicine. Microbiol Mol Biol Rev.

[4] Laval K, Vernejoul JB, Van Cleemput J, Koyuncu OO, Enquist LW (2018). Virulent Pseudorabies Virus Infection Induces a Specific and Lethal Systemic Inflammatory Response in Mice. J Virol.

[5] Avak S, Bienzle U, Feldmeier H, Hampl H (1987). Habermehl K-OJTL. Pseudorabies in man.

[6] Ai JW, Weng SS, Cheng Q, Cui P, Li YJ, Wu HL (2018). Human endophthalmitis caused by pseudorabies virus infection, China, 2017. Emerg Infect Dis.

[7] Yang H, Han H, Wang H, Cui Y, Liu H, Ding S (2019). A case of human viral encephalitis caused by pseudorabies virus infection in China. Front Neurol.

[8] Li A, Lu G, Qi J, Wu L, Tian K, Luo T (2017). Structural basis of nectin-1 recognition by pseudorabies virus glycoprotein D. PLoS Pathog.

[9] Fan S, Yuan H, Liu L, Li H, Wang S, Zhao W (2020). Pseudorabies virus encephalitis in humans: a case series study. J Neurovirol.

[10] Liu Q, Wang X, Xie C, Ding S, Yang H, Guo S (2021). A novel human acute encephalitis caused by pseudorabies virus variant strain. Clin Infect Dis.

[11] Yang X, Guan H, Li C, Li Y, Wang S, Zhao X (2019). Characteristics of human encephalitis caused by pseudorabies virus: a case series study. International journal of infectious diseases : IJID : official publication of the International Society for Infectious Diseases.

[12] Hu F, Wang J, Peng XY (2021). Bilateral necrotizing retinitis following encephalitis caused by the pseudorabies virus confirmed by next-generation sequencing. Ocul Immunol Inflamm.

[13] Yan W, Hu Z, Zhang Y, Wu X, Zhang H (2021). Case report: metagenomic next-generation sequencing for diagnosis of human encephalitis and endophthalmitis caused by pseudorabies virus. Front Med.

[14] Ying M, Hu X, Wang M, Cheng X, Zhao B, Tao Y (2021). Vitritis and retinal vasculitis caused by pseudorabies virus. J Int Med Res.

[15] Liu Y, Yang B, Bai R, Wang Y, Li J, Ren H (2022). The clinical characteristics of human pseudorabies virus infection: a case report and literature review. Chin J Neurol.

[16] Yue L, Yi L, Fei T, MengWu T, Man L, LiQing W (2022). Human encephalitis complicated with ocular symptoms associated with pseudorabies virus infection: a case report. Front Neurol.

[17] Spear PG, Eisenberg RJ, Cohen GH (2000). Three classes of cell surface receptors for alphaherpesvirus entry. Virology.

[18] Zhou Y, Nie C, Wen H, Long Y, Zhou M, Xie Z (2022). Human viral encephalitis associated with suid herpesvirus 1. Neurol Sci.

[19] Wang D, Tao X, Fei M, Chen J, Guo W, Li P (2020). Human encephalitis caused by pseudorabies virus infection: a case report. J Neurovirol.

[20] Kopp SJ, Karaba AH, Cohen LK, Banisadr G, Miller RJ, Muller WJ (2013). Pathogenesis of neonatal herpes simplex 2 disease in a mouse model is dependent on entry receptor expression and route of inoculation. J Virol.

[21] Kopp SJ, Banisadr G, Glajch K, Maurer UE, Grünewald K, Miller RJ (2009). Infection of neurons and encephalitis after intracranial inoculation of herpes simplex virus requires the entry receptor nectin-1. Proc Natl Acad Sci U S A.

[22] Karaba AH, Kopp SJ, Longnecker R (2012). Herpesvirus entry mediator is a serotype specific determinant of pathogenesis in ocular herpes. Proc Natl Acad Sci U S A.

[23] Edwards RG (2017). Longnecker RJJov. Herpesvirus entry mediator and ocular herpesvirus infection: more than meets the eye.

[24] Zhu S, Viejo-Borbolla A (2021). Pathogenesis and virulence of herpes simplex virus. Virulence.

[25] De Tiege X, Rozenberg F, Des Portes V, Lobut J, Lebon P, Ponsot G (2003). Herpes simplex encephalitis relapses in children: differentiation of two neurologic entities.

[26] Skoldenberg B, Aurelius E, Hjalmarsson A, Sabri F, Forsgren M, Andersson B (2006). Incidence and pathogenesis of clinical relapse after herpes simplex encephalitis in adults. J Neurol.

[27] Gutekunst D, Pirtle E, Miller L (1980). Stewart WJAjovr. Isolation of pseudorabies virus from trigeminal ganglia of a latently infected sow.

[28] Rassnick S, Enquist LW, Sved AF (1998). Card JPJJov. Pseudorabies virus-induced leukocyte trafficking into the rat central nervous system.

[29] Bradshaw MJ, Venkatesan A (2016). Herpes Simplex Virus-1 Encephalitis in Adults: Pathophysiology, Diagnosis, and Management. Neurotherapeutics.

