# Supplementary material for: A case report of long-delayed diagnosis of pseudorabies virus encephalitis with endophthalmitis: lessons from metagenomic next generation sequencing
Source: BMC Neurol. 2023 May 16;23:192. doi: 10.1186/s12883-023-03227-1 (PMC10186779; doi:10.1186/s12883-023-03227-1)
Supplement: Supplementary file 1 — Additional file 1. [file 12883_2023_3227_MOESM1_ESM.docx]

| Characteristics | Result | reference range |
| --- | --- | --- |
| White blood cell(WBC) | 8.60 | 3.50-9.50 10^9/l |
| Percentage of neutrophils | 84.70 | 50.00-70.00% |
| Hypersensitive C reaction | 0.50 | 0-5.00mg/l |
| Calcitonin original | negative |  |
| Electrolyte | negative |  |
| Blood glucose | 7.8 | <11.1mmol/l |
| Glutamic-pyruvic transaminase | 47.00 | 5.00-40.00u/l |
| Aspertate aminotransferase | 55.00 | 8.00-40.00u/l |
| Hepatitis B antigens | 0.00 | <0.05IU/ml |
| Hepatitis C antibodies | 0.02 | <1.0 S/CO |
| Syphilis antibodies | 0.01 | <1.0 S/CO |
| HIV antibodies | 0.08 | <1.0 S/CO |
| TORCH |  |  |
| Rubella virus | IgG- negative |  |
|  | IgM- negative |  |
| Epstein-Barr virus | IgG- negative |  |
|  | IgM- negative |  |
| Herpes simplex virus | IgG- negative |  |
|  | IgM- negative |  |
| Cytomegalovirus | IgG- negative |  |
|  | IgM- negative |  |
| Toxoplasma | IgG- negative |  |
|  | IgM- negative |  |
| Antibodies of autoimmune encephalitis* |  |  |
| Serum | negative |  |
| CSF | negative |  |

Supplemental Table 1.Detailed results laboratory findings of the patient

* Antibodies of autoimmune encephalitis including NMDAR IgG, AMPAR1 IgG, AMPAR2 LgG, LGI1 IgG, CASPR2 IgG, GABAR IgG, DPPX IgG, IgLON5 IgG, Glyα1 IgG, mGluR5 IgG, D2R IgG and GAD65 IgG.
